# What, if anything, can be considered an amodal sensory dimension?

**DOI:** 10.3758/s13423-023-02447-3

**Published:** 2024-02-21

**Authors:** Charles Spence, Nicola Di Stefano

**Affiliations:** 1 Department of Experimental Psychology, New Radcliffe House, University of Oxford, Oxford, OX2 6BW UK; 2 Crossmodal Research Laboratory, University of Oxford, Oxford, UK; 3 Institute of Cognitive Sciences and Technologies, National Research Council of Italy (CNR), Rome, Italy

**Keywords:** Amodal, Crossmodal correspondences, Universal dimensions, Amodal completion, Amodal relations, Amodal representations

## Abstract

The term ‘amodal’ is a key topic in several different research fields across experimental psychology and cognitive neuroscience, including in the areas of developmental and perception science. However, despite being regularly used in the literature, the term means something different to the researchers working in the different contexts. Many developmental scientists conceive of the term as referring to those perceptual qualities, such as, for example, the size and shape of an object, that can be picked up by multiple senses (e.g., vision and touch potentially providing information relevant to the same physical stimulus/property). However, the amodal label is also widely used in the case of those qualities that are not directly sensory, such as, for example, numerosity, rhythm, synchrony, etc. Cognitive neuroscientists, by contrast, tend to use the term amodal to refer to those central cognitive processes and brain areas that do not appear to be preferentially responsive to a particular sensory modality or to those symbolic or formal representations that essentially lack any modality and that are assumed to play a role in the higher processing of sensory information. Finally, perception scientists sometimes refer to the phenomenon of ‘amodal completion’, referring to the spontaneous completion of perceptual information that is missing when occluded objects are presented to observers. In this paper, we review the various different ways in which the term ‘amodal’ has been used in the literature and the evidence supporting the various uses of the term. Morever, we highlight some of the various properties that have been suggested to be ‘amodal’ over the years. Then, we try to address some of the questions that arise from the reviewed evidence, such as: Do different uses of the ‘term’ refer to different domains, for example, sensory information, perceptual processes, or perceptual representations? Are there any commonalities among the different uses of the term? To what extent is research on cross-modal associations (or correspondences) related to, or can shed light on, amodality? And how is the notion of amodal related to multisensory integration? Based on the reviewed evidence, it is argued that there is, as yet, no convincing empirical evidence to support the claim that amodal *sensory* qualities exist. We thus suggest that use of the term amodal would be more meaningful with respect to abstract cognition rather than necessarily sensory perception, the latter being more adequately explained/understood in terms of highly redundant cross-modal correspondences.

**Supplementary Information:**

The online version contains supplementary material available at 10.3758/s13423-023-02447-3.

## Are there amodal sensory dimensions?

Over the last century or so, a number of researchers have argued for the existence of various putatively amodal stimulus dimensions. Literally meaning ‘without’ modality (see Bahrick, 2010), the term ‘amodal’ is often taken to mean that the same information can be picked up regardless of the sensory source, or modality, by which that information was acquired (Walker-Andrews, 1994). For instance, the results of an early psychophysical study by Von Hornbostel (1931) led to the claim that intensity might be a universal dimension of sensory experience (see also Hayek, 1952, p. 21).^1^

Developmental scientists have long been intrigued by the possibility that ‘amodal relations’ may help to scaffold multisensory development (see Bremner et al., 2012). However, a closer look at the developmental literature soon reveals that at least three different (and qualitatively distinct) definitions of amodal relations have been adopted by those researchers working in this area (see Table 1). One use of the term is when referring to those perceptual attributes, such as, for example, the size and shape of an object, that are considered to be amodal because multiple senses (vision and touch) can potentially provide information relevant to the same physical stimulus/property (cf. Streri et al., 1993). The second definition of amodal refers to those sensory attributes (or qualities) such as, for example, stimulus intensity, that can putatively be picked up by each and every sense, even though this perceptual quality is itself typically associated with different kinds of physical energy/chemical stimulation. A third use of the term ‘amodal’ by developmental researchers relates to those judgements that are not directly (or at least not necessarily) sensory, such as, for example, those relating to numerosity, rhythm, synchrony, etc.

**Table 1:** Summary of the different ways in which the term 'amodal' has been used in the literature

Research area/usage	Those using the term in this way/context	Comments/problems with this interpretation
**Developmental psychology**		
**(Amodal relations)**		
Physical property that can be	Aristotle (1907); Gibson (1979);	Researchers do not agree on
picked-up by more than	Lewkowicz & Turkewitz (1980);	whether properties detectable by
one sense (e.g., shape, size)	Gogate & Bahrick (1998)	two or more senses, or by all of the senses
Phenomenal quality associated	Von Hornbostel (1931); Werner (1934);	Evidence suggests ratio based
with stimuli in every sense	Lewkowicz & Turkewitz (1980);	comparison of unisensory
(e.g., intensity)	Walker-Andrews (1994); Bahrick (2010)	prothetic dimensions (see Stevens, 1957)
Non-sensory mental category (e.g., rhythm, numerosity)	Lewkowicz & Turkewitz (1980); Bahrick (2010)	Note that these are not sensory properties, but more like mental categories
**Cognitive neuroscience**		
** (Cognitive process)**		
Central cognitive process (or representation)/non-sensory property	Arnell (2006); Simpson (1996); Tamber-Rosenau et al. (2013); Van Wassenhove (2009)	Unclear relation to sensory properties
**Perception research**		
**(Amodal completion)**		
Representation of parts/qualities of the perceived object that observers get no direct sensory stimulation from	Kanizsa & Gerbino (1982); Kim et al. (2014); Nanay (2018); Gerbino (2020)	Based on definition of 'amodal' as meaning without modality

The notion of amodality has recently been discussed with respect to the nature of concepts, with amodalists (i.e., those who argue that concepts are represented by amodal symbols) and modalists (i.e., those who see concepts as involving crucially representations including sensorimotor information) claiming that the same empirical evidence is compatible with their views instead (Michel, 2021). Cognitive neuroscientists use the term amodal when referring to those cognitive processes that lie between perceptual input and motor output and that do not have an obvious sensory component (see also Fowler, 2004, on the notion that speech perception can be considered as a supramodal or amodal phenomenon).^2^ Finally, many perception scientists have long been interested in the topic of ‘amodal completion’, though, as we will see later, this usage of the term amodal is by no means unproblematic either.

In this narrative historical review (see Ferrari, 2015, and Furley & Goldschmied, 2021, on the narrative style of literature review), we highlight the three distinct areas in which the term ‘amodal’ has been used in the literature over the last century, namely developmental science, cognitive neuroscience, and perception science. We expand on the question that forms the title of this review while also exploring related sub-questions, such as: Do different uses of the ‘term’ refer to different domains, for example, sensory information, perceptual processes, or perceptual representations? Is the amodal concept exclusively perceptual, or does it also extend (or refer) to some cognitive/conceptual ability? Are certain putatively amodal perceptual relations better conceptualized in terms of highly redundant cross-modal correspondences (see Spence, 2011; Spence & Di Stefano, 2023)? What exactly is amodal about ‘amodal completion’? And is there a common element in the different uses of the term amodal across developmental science, cognitive science, and perception research? However, before addressing these questions, and the various contemporary uses of the term amodal, we first take a brief look at historic accounts of the unity of the senses that allegedly constitute the theoretical grounding for some modern views of the topic (e.g., Marks, 1978).

## Early considerations on universal properties

### Aristotle on the separation/integration of the senses

More than two millennia ago, Aristotle distinguished between the special objects of perception and common sensibles. According to his theory of perception, each sense has its special objects, “which cannot be perceived by any other sense than that one in respect of which no error is possible; in this sense color is the special object of sight, sound of hearing, flavor of taste” (Aristotle, 1907, p. 418a). The special objects of perception contrast with common sensibles, which are features of the world that can be perceived in their own right by different senses: “For the perception of magnitude, figure, roughness, smoothness, and sharpness and bluntness, in solid bodies, is the common function of all the senses, and if not all, then at least the common function of sight and touch” (Aristotle, 1906, 442b; see also Aristotle, 1907, 418a10–11, 19). In Western philosophy, early conceptualizations of the senses tend to stress the distinction between different sensory modalities, while, at the same time, also emphasizing the intimate link between them. For instance, Aristotle presented the *sensus communis* as a putative psychological function connecting sensory impressions gathered from the five senses and processing them as a whole (Aristotle, 1907; see Johnstone, 2021).

Aristotle’s distinction between special objects of perception and common sensibles would appear to be reflected in the Gibsonian account of perception. In one passage of his foundational work, *The senses considered as perceptual systems*, J. J. Gibson considers the example of a fire, whose perceptual presence is provided by four sources of information, namely sound, odour, heat, and light. Gibson observes that “Different stimulus energies – acoustical, chemical, radiant – can all carry the same stimulus information […] patterns in the flux of sound, touch, and light from the environment may be equivalent to one another by invariant laws of nature” (Gibson, 1966, p. 55). Elsewhere, Gibson claimed that there are also kinds of sensory information that are essentially transmitted by (or available to) one and only one sense: “The material color (pigmentation) of a surface is not tangible but only visible. The relative temperature, however, is tangible but not visible” (Gibson, 1966, p. 123). Interestingly, in addition to most commonly offered arguments consistent with special objects and common sensibles, Gibson seemingly suggests that certain specific types of information exist exclusively as emergent, higher-order relations between patterns of information available to specific sensory systems, namely vestibular and somatosensory (Gibson, 1966, pp. 59-75; see also Stoffregen et al., 2017).^3^

The views that have been put forward by both Aristotle and Gibson suggest that, beyond the naïvely accepted distinction between different sensory channels and kinds of information, one must also consider amodal dimensions that require some kind of unity of the senses.^4^ The latter suggestion would appear to imply that some common, or shared, mechanism (e.g., the Aristotelian *sensus communis*) is evoked to process such amodal sensory data and/or to generate perceptual knowledge from amodal inputs (see also Inderelst, 2017). Taken together, these considerations open up several different lines of academic inquiry, from focusing on the implication of the existence of amodal dimensions for sensory processing through to asking whether and how such amodal or universal dimensions are related to unimodal and cross-modal perception.

### **U**nity of sensations and the universal dimensions of perceptual experience

Almost a century ago, Erich Moritz von Hornbostel argued for the unity of the senses.^5^ As the Austrian ethnomusicologist and psychologist pointedly put it: “It matters little through which sense I realize that in the dark I have blundered into a pigsty” (1927, p. 83). A few years later, the eminent psychologist Heinz Werner seemingly echoed Aristotle when he wrote that: “Le lien intime des sens, l’existence de qualités intersensorielles comme la clarté, l’intensité, la rugosité, etc., tout cela est fondé sur le fait que l’organisme psycho-physique réagit dans sa totalité, avant toute séparation en sphères distinctes de sensibilité” (Werner, 1934, p. 202). This statement can be translated as: “The senses’ intimate link – the existence of intersensory qualities such as brightness, intensity, roughness, etc. – all of this is based on the fact that the psychophysical organism reacts as a whole, before any separation into distinct spheres of sensitivity”.^6^ The latter suggestion, it should be noted, seemingly runs counter to the large body of contemporary neuroscientific evidence highlighting the segregation of sensory inputs at the earliest stages of information processing (e.g., see Bremner et al., 2012; Calvert et al., 2004; Stein, 2012). The interested reader is directed to Felleman and Van Essen’s (1991) authoratitive review of the highly distributed and hierarchical nature of sensory information processing in primate cerebral cortex. Furthermore, it is worth noting how psychophysicists would seem to consider auditory loudness and visual brightness to be cross-modally corresponding dimensions rather than as tapping into the same unitary amodal dimension of stimulus intensity (e.g., see Stevens, 1966; Stevens & Marks, 1965, 1980, for cross-modality matching of brightness and loudness). Similarly, and somewhat confusingly, the most natural cross-modal correspondence with visual brightness is not with auditory brightness (Siedenburg et al., 2021), again arguing against an amodal brightness (or intensity) dimension.

### Amodality and the psychophysics of cross-modal matching

One of the earliest empirical studies on putatively amodal perceptual dimensions was published by Von Hornbostel (1931). He believed that brightness represented ‘a universal dimension of sensory experience’. To support this idea, Von Hornbostel conducted a study in which three participants had to match sounds of different pitches to points along a greyscale, and to cross-modally match scents with greyscale values. Given the apparent transitivity between different cross-modal comparisons that Von Hornbostel’s results revealed, his findings were taken to be consistent with the concept of ‘sensory brightness’ being common to all of the senses, and hence a universal dimension of sensory experience. (We take the latter to mean that Von Hornbostel was referring to sensory attributes that share the same phenomenal quality.) However, although the results obtained may support robust cross-modal psychophysics, it is worth noting that the cross-modal match may not be something that people are themselves subjectively confident of. As Von Hornbostel (1927/1938, pp. 210-211) wrote almost a century ago: “Anyone can find on the piano that tone which sounds as bright as lilac smells. (Generally he thinks the task nonsense at first, but, if he can be persuaded to deal with such nonsense at all, it goes very well.)” (see also Postnova et al., 2020).

However, early objections were raised against the existence of amodal stimulus dimensions. For instance, the North American psychologist Cohen (1934) argued that these cross-modal mappings were actually better conceptualized as relative/relational judgements instead (i.e., rather than necessarily a cross-modal perceptual mapping of brightness based on amodal properties; cf. Hartshorne, 1934, for an extended discussion on this theme). According to Cohen, Von Hornbostel’s experimental stimuli were ‘analogous’ rather than ‘identical’. The Harvard psychologist explained as follows: “It would not be unreasonable then to suppose that cross-modality comparison should be based (physiologically, if not introspectively) upon relative positions within different ‘absolute’ scales. According to this view equation with respect to brightness of two experiences of different modalities would involve nothing more than the identity of relative positions upon two wholly independent scales.” (Cohen, 1934, p. 119). Despite refuting the idea of amodal stimulus dimensions, this explanation must assume that the two ‘absolute’ scales are commensurable, at least with respect to very general properties, such as dimensionality.

A little over half a century later, Mellers and Birnbaum (1982, p. 593) made the point clearer when they distinguished between mapping theory and relation theory for explaining cross-modality matching: “According to mapping theory, psychological values of stimuli from different continua are mapped onto a common scale of sensation and can be compared directly. A cross-modality match is presumed to occur when equal strength sensations are elicited by stimuli on different continua. According to relation theory, relationships (e.g., ratios) between pairs of stimuli from different continua are compared. In physical measurement, a mass in grams cannot be compared with a length in centimeters but ratios of masses can be compared with ratios of length. By analogy, it may be possible to compare the ratio of the heaviness of two weights to the ratio of the loudness of two tones, since the ratios of stimulus pairs are on a common scale.” (see also Teghtsoonian, 1980; Teghtsoonian & Teghtsoonian, 1965, 1970).

Mellers and Birnbaum’s (1982) reference to ‘different continua’ presumably supports the view of modality-specific (though presumably comparable) perceptual continua rather than supporting an amodal account in this particular case. Later, Mellers and Birnbaum (1982, p. 600) suggest that: “In cross-modality judgments, the scale values are influenced by the stimulus distribution: It appears that subjects compare the relative position of a stimulus in its distribution with the relative position of a stimulus of another modality to its distribution.” In other words, their results were taken to support a psychological relativity theory of cross-modal judgements. Ultimately, therefore, one is left wondering whether the robust psychophysics (e.g., of transitivity) obtained when comparing judgements across various pairs of senses (see Ellermeier et al., 2021, on the ratio-based cross-modal matching of visual brightness and sound intensity; cf. Heller, 2021; Luce et al., 2010) reflects anything more than merely the application of ratio properties within qualitatively distinct unimodal prothetic dimensions (see Cohen, 1934; Root & Ross, 1965; Stevens, 1957, 1966, 1971; Stevens & Guirao, 1963). Regarding this point, Marks, Hammeal, and Bornstein (1987, p. 34) observed that: “As a general rule, psychophysicists who study crossmodal matching have concerned themselves primarily with determining precise quantitative measures of intersensory equivalence; their purpose is usually to test theoretical predictions made from psychophysical functions (which relate judgements of sensory magnitudes to physical intensities) derived for individual continua like loudness and brightness.” Thus, while the robust psychophysics of cross-modal matching is undoubtedly consistent with the existence of an underlying amodal dimension guiding people’s responses, it certainly doesn’t necessarily entail it.

Having briefly illustrated the relevance of the topic throughout Western thought and across different disciplines, we now go on to more clearly distinguish among the different uses of the term, namely amodal relations, amodal cognitive processes, and amodal completion, each one mainly relevant to a different field of research, i.e., developmental psychology, cognitive neuroscience, and perception science, respectively. We then try to evidence the overlap or similarities among those occurrences, if any, and focus especially on the conceptual difficulties that arise from the literature that is reviewed.

## Developmental science: The intersensory redundancy hypothesis and amodal relations

The putative existence of amodal dimensions has occasionally been considered as an inspirational principle for research in developmental psychology, with Linda Smith (1987, p. 96) once suggesting that: “We have a lot to gain if we can get beyond mapping specific dimensions one to another and instead delineate the amodal dimensions.” Indeed, many developmental psychologists are firmly of the belief that intersensory redundancy, of which amodal relations can be considered as but one example, provide a fundamental role in helping to scaffold the subsequent multisensory interactions that emerge during human development (e.g., Bahrick, 2010; Bahrick et al., 2004; Bahrick & Pickens, 1994; Lickliter & Bahrick, 2012; Nava et al., 2017; Slater et al., 1999; Smith, 1987). According to Gibson’s (1979) ecological theory of perception, amodal information, that is information that is not specific to any one sensory modality and that can thus be conveyed redundantly across several sense modalities simultaneously, is obtained directly from adaptive interaction between an organism and its environment.

Based on a large body of experimental research on the role of putatively amodal characteristics in the development of early perceptual abilities, Bahrick and Lickliter (2000) formulated the ‘intersensory redundancy hypothesis’ (IRH) to explain how it is that infants perceive coherent, unified multisensory stimuli and events. In particular, according to Bahrick (2010, p. 44): “Properties of objects and events such as temporal synchrony, rhythm, tempo, duration, intensity, and co-location are common across auditory, visual, and proprioceptive stimulation.” (see Table 2 for a summary of putatively amodal dimensions). The theory proposes that, in order to be perceptually integrated, the same information must be spatially coherent (though see Spence, 2013, for evidence suggesting that this popular claim is often not true in the case of adult multisensory perception, and Spelke, 1976, for similar evidence in 4-month-old infants). Furthermore, according to the theory, the information should also be temporally synchronous across two or more senses, and cross-sensory integration is thus only possible for amodal properties that are not specific to a single sense modality (e.g., shape, rhythm, duration, and intensity). In other words, regardless of the sensory modality that is solicited, similar qualities are invariants in the environment perceived as originally integrated piece of information. According to the IRH, sensitivity to amodal properties allows young infants’ attention to be selectively directed (i.e., in an exogenous manner) to unitary and meaningful events in their environment (Bahrick, 1992). Here, we argue that while a number of those dimensions commonly considered by the developmental psychologists to be amodal do indeed exhibit a high degree of intersensory redundancy, one might want to stop short of asserting that this necessarily means that they are in any meaningful sense amodal (see also Jones, 1981).

**Table 2:** Summary of the various properties that have been suggested to be amodal over the years

			Stimulus-related					Temporal					Spatial		
Study / Source	Area(s)	Meaning of amodal	Intensity	Thickness	Texture	Flexibility	Brightness	Rate	Duration	Tempo	Synchrony	Rhythm	Location	Extent/Size	Shape
Aristotle (1907)	Perception research	Physical property that can be picked-up by more than one sense	*		*										*
Moul (1930)	Perception research	Property associated with stimuli in every sense		*											
Von Hornbostel (1931); cf. Cohen (1934)	Perception research	Property associated with stimuli in every sense					*								
Werner (1934)	Perception research	Physical property that can be picked-up by more than one sense	*		*		*								
Lewkowicz & Turkewitz (1980)	Developmental science	Non-sensory mental category	*					*	*			*	*	*	*
Walker-Andrews (1994)	Perception research	Property associated with stimuli in every sense	*		*	*			*					*	
Bahrick (2010)	Developmental science	Non-sensory mental category	*						*	*	*	*	*		

According to Lewkowicz and Turkewitz (1980, p. 597): “Intensity, rate, duration, spatial location, spatial extent, rhythm, and shape all represent amodal features of the world that can be specified in more than one modality. They stand in distinction to such modality specific features of stimulation as redness, sweetness, and pitch.” Meanwhile, according to Walker-Andrews (1994, p. 42): “Amodal properties (size, texture, flexibility, duration, and intensity) ... may be picked up by any modality”.^7^ However, as will be highlighted below, it can be argued that the various examples of amodal relations that have been put forward in the literature by developmental psychologists to date (as highlighted in Table 2) are actually of three qualitatively different types, or kinds, namely cross-modal relations, amodal relations, and amodal mental categories.

### Absolute versus relative cross-modal relations

Part of what the notion of amodal relations buys one, when thinking in a developmental context, is the idea that cross-modal relations are absolute rather than relative. According to Smith (1987, pp. 97-98): “This suggestion of a trend from dichotomous, categorical treatments of continua to more relativistic ones ought not to be confused with the issue of absolute versus relative correspondences across dimensions. The existence of absolute correspondences between dimensions would mean that particular values on one dimension map onto particular values on another – for example, higher is not like brighter; rather, a specific pitch would match a specific brightness. As Marks et al. point out though, there is little evidence for such absolute correspondences.” That said, Bahrick and Pickens (1994) talk of invariant amodal relations. It is presumably more useful to have an absolute mapping between sensory inputs rather than one that is relative, and thus dependent on the context (i.e., and any other stimuli that may be presented at around the same time). Relevant here, though, the vast majority of pitch-based cross-modal correspondences have been found to be relative (see Spence, 2019; Spence & Di Stefano, 2022b).

### Amodal relations

According to another definition, perceptual attributes such as, for example, the size and shape of an object, are considered amodal because multiple senses (vision and touch) potentially provide information relevant to one and the same physical stimulus/property. This, it should be noted, is subtly different from von Hornbostel’s (1927) notion of universal dimensions of perceptual experience.^8^ The emphasis in the latter case would appear to be on the perceptual experience itself (i.e., ‘what it is like’), whereas the emphasis for many of the amodal dimensions that have been proposed previously has been on the multiple routes to picking-up information about physical properties ‘out there’, regardless of the perceptual qualities (or phenomenal feel) that may be associated with that information. However, other sensory attributes, such as, for example, stimulus intensity, are considered to be amodal by developmental psychologists because the same phenomenal quality is associated with the information putatively picked up by multiple senses, even though it is associated with different kinds of modality-specific attributes/stimulation. Some commentators have suggested that the same dimension, such as sensory intensity (Lewkowicz & Turkewitz, 1980), sensory brightness (Von Hornbostel, 1931; though see Cohen, 1934; Hartshorne, 1934), and even sensory ‘thickness’ (Moul, 1930) can be considered as amodal dimensions, given that these perceptual qualities can be associated with two or more (and possibly all of the) senses.

Amodal sensory concepts are conceived of as the same physical property (such as shape) being picked up via multiple senses (see Lewkowicz & Turkewitz, 1980). By contrast, according to Spence and Di Stefano (2023), such cross-modal relations may actually be more appropriately conceptualized as reflecting highly-redundant cross-modal correspondences (cf. Parise & Spence, 2013) rather than necessarily supporting the existence of amodal sensory dimensions.^9^ In summary, the concept of amodal relations is important to developmental psychologists inasmuch as it provides a framework for thinking about multisensory development (Bremner et al., 2012).

In addition to the early objections of the universal correspondences account that have been mentioned already, a number of additional issues have also been raised in the literature regarding the status of amodal sensory properties. On the one hand, there would appear to be disagreement about how exactly amodal dimensions should be defined. Writing that common sensibles are “perceptible by any and all of the senses” (Aristotle, 1907, p. 418a), Aristotle left it somewhat unclear as to whether these common sensibles should be common to all of the senses or just to two or more of them (see also Bahrick, 2010; Gogate & Bahrick, 1998; Walker-Andrews, 1994). Aligning with most commentators’ interpreation (e.g., Knuuttila, 2008), Johnstone (2021) takes Aristotle’s considered view to have been that common sensibles are perceptible in their own right by more than one sensory modality, but need not necessarily be perceptible by all five of the commonly accepted senses.

### Amodal mental categories

A number of developmental researchers have also chosen to describe as amodal those attributes such as, for example, numerosity, rhythm, and synchrony, that are not themselves directly sensory (see Table 2). Several studies demonstrated that people can recognize the similarity between temporal patterns no matter whether the stimulus sequence is heard, felt, or seen (e.g., Fraisse, 1981; Marks, 1987a, b; see also Frings & Spence, 2010), thus suggesting that the neural mechanisms enabling the processing of temporal patterns may not themselves be modality-specific, and hence that temporal pattern is likely to be an amodal stimulus property.^10^ Relevant here, Huang et al. (2012) conducted a study in which their participants had to perform a rhythm discrimination task in which rhythmic patterns were presented either unimodally (i.e., to the auditory or tactile modality) or else cross-modally (i.e., composed of a sequence of auditory and tactile stimuli). Although the participants were able to discriminate the rhythms at above-chance levels in all conditions, when presented auditorily, their performance was found to be somewhat more accurate. Note that similar findings have been reported in the literature on visual and auditory rhythm perception (e.g., see Grahn, 2012; McAuley & Henry, 2010). Taken together, such results would appear to support Lewkowicz and Turkewitz’s (1980) claim that rhythm can be considered to be amodal (at least according to this particular definition of amodal), as well as an earlier claim by E. J. Gibson (1969), who considered temporal (and spatial) dimensions as available to all sensory modalities from birth onward.^11^

A similar argument has been made with respect to the putatively amodal nature of judgements of numerosity (e.g., see Féron et al., 2006; Gallace et al., 2007; Kobayashi et al., 2004; Togoli & Arrighi, 2021; cf. Nuerk et al., 2005; Piazza et al., 2006). It is, however, important to recognize that both numerosity and rhythm are linked to the grouping of stimuli, and have no meaning when referred to isolated stimuli because they are not themselves sensory properties of the stimuli concerned (i.e., in the way that stimulus intensity is, say).^12^ In fact, while it seems possible to conceptualize a given numerosity without necessarily having any sensory content, it would intuitively seem much more difficult, if not impossible, to represent or mentalize a given stimulus intensity without also considering a particular sensory stimulus/input. To put this in Kantian terms, features such as numerosity and rhythm can be considered as (mental/formal) categories that essentially serve to help organize perceptual materials, i.e., phenomenal appearances, but they are not perceptual in nature and thus are not perceived directly.^13^ On the contrary, redness or sweetness are sensory features that can only be directly perceived, or apprehended, by means of the senses (Kant, 1998).

Interestingly, comparative research has convincingly shown that many of the categories that have been considered as amodal in humans are widespread in the animal kingdom as well. For instance, Vallortigara et al. (2010) reviewed various findings demonstrating different degrees of “protomathematical” abilities in monkeys, rats, salamanders, and chicks. Meanwhile, Rodríguez et al. (2015) demonstrated that spiders (‘*nephila clavipes*’) keep track of captured prey counts in a way that demonstrate an ability to discriminate numerosity versus other quantities (e.g., the mass of prey; see also Carazo et al., 2009), while Bortot et al. (2019) have demonstrated that honeybees rely on absolute rather than relative numerosity in number discrimination. When taken together with similar evidence collected in human infants (see Butterworth, 2005, for a review), these findings suggest that a minimum core knowledge of numerosity would be spontaneously (innately) present in both animals and human infants, with completely different sensory systems, including invertebrates. In other words, the mere existence of amodal categories (e.g., numerosity) in animals that have completely different sensory systems seemingly suggests that at least that specific category (i.e., numerosity) cannot be explained exclusively in terms of sensory information/processing. In evolutionary terms, the universal ability to understand numbers must have provided an invaluable tool to survival, shaped through natural selection to best suit the needs of the different species (Vallortigara et al., 2010).^14^

### Is complete redundancy ever really acheived across the senses?

Going back again to one of the key early questions that was posed by Aristotle, one might ask “why we have more senses than one” (Aristotle, 1907, p. 425b). Aristotle’s answer was that multiple senses exist “to prevent a failure to apprehend the common sensibles […] The fact that the common sensibles are given in the objects of more than one sense reveals their distinction from each and all of the special sensibles” (Aristotle, 1907, p. 425b). That is, Aristotle argued that multiple, distinct senses must exist so that we may distinguish those percepts that are redundant across the senses from those that are unique to a particular sense. Gogate and Bahrick (1998, p. 99) seemingly echo Aristotle’s position when they write that: “Amodal information is information which is completely redundant across two or more senses”. Here, though, it is important to note that there is virtually never perfect redundancy between the senses, even when multiple senses are potentially capable of picking up on the same environmental property, such as, for example, size/shape (Spence et al., 2013; cf. Huang et al., 2012, discussed earlier), the precision/accuracy of different unisensory estimates rarely aligns perfectly (Ernst & Banks, 2002). Consider here how vision and touch only pick up on the same shape/size information over a very narrow range of stimulus dimensions. What is more, individual sensory estimates nearly always differ in their precision/accuracy, so are virtually never perfectly redundant. Meanwhile, one might also consider how auditory, and even olfactory, cues provide information (albeit more or less precise/accurate) to size/shape (see Evans & Treisman, 2010; Gallace & Spence, 2006; Ngo et al., 2013; Spence & Zampini, 2006).

**Fig. 1:**
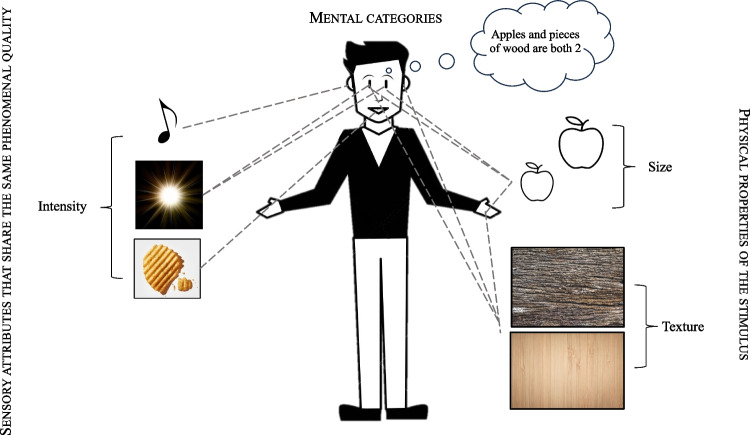
The three different categories in which the reviewed occurrences of the term ‘amodal’– namely amodal relations, amodal mental categories, and amodal cognitive processes – can be grouped. (1) *Physical properties* of the stimulus are those features that characterize the perceptual quality of the stimulus and that can be picked up by different senses, such as size, shape, and texture. (2) *Sensory attributes* are attributes that define the quality of sensory information/experience across the senses, such as the intensity, say, of sound, light, and taste, and which share the same phenomenal quality. (3) *Amodal mental categories* refer to those abstract concepts that can be applied to sensory objects or percepts across different modalities, such as numerosity

In terms of roughness perception (i.e., what is commonly referred to as texture), meanwhile, it would seem as though in the case of vision and touch the physical information that is picked up is the same as is the phenomenal quality (see Fig. 1). However, when researchers talk about auditory roughness, both the physical information that is picked up and its phenomenal quality differs from the case of visual and tactile/haptic roughness, this despite the fact that physical contact with rough surfaces typically gives rise to interaction sounds that are themselves described as sounding rough (see Di Stefano & Spence, 2022, for a recent review).

### Interim summary

Over the last half century or so, developmental researchers have used the term ‘amodal’ in at least three different ways. Several of the amodal features that have been put forward by the developmental researchers related to the three qualitatively different kinds of amodality that have been outlined here. The dimensions that Bahrick (2010) and others choose to include as examples of amodal (see Table 2 for a summary) consist of a mixture of those that are amodal by virtue of the fact that different senses sometimes happen to be able to pick up the same sensory information, such as vision and touch providing information about the size and shape of hand-held objects (in such cases, there is seemingly no explicit necessity for the relevant unisensory experiences to be phenomenally similar; Ernst & Banks, 2002),^15^ or in virtue of the fact that the perceptual/phenomenal quality is somehow equivalent, as in the case of stimulus intensity (despite the fact that what is being picked up is in some sense different between each sense; i.e., the physical stimulus is different). Finally, we have mental categories such as rhythm, tempo, rate,^16^ etc. also being classed as amodal because while these descriptors can be applied to collections of sensory stimuli, they do not depend on sensory stimulation.

## Amodal cognitive processes

The second broad usage of the term ‘amodal’ has been by those cognitive neuroscientists wanting to describe putative central cognitive processes that are not linked to a specific modality. The term is used by researchers working in this area to describe the operation of central information processing, such as, for example, the attentional bottleneck that is postulated to be responsible for the ‘attentional blink’ (e.g., Arnell, 2006; Potter et al., 1998; Simpson, 1996; Tamber-Rosenau et al., 2013; van Wassenhove, 2009). Meanwhile, Van Wassenhove uses the term in relation to the nature of temporal representation in perception, addressing the issue of “whether time perception should be conceived of as being tightly coupled to a sensory modality or whether the representations for time perception acquire a level of abstraction” (van Wassenhove, 2009, p. 1818). Van Wassenhove suggests that the temporal content of perception can be read through different systems and that it involves amodal representation (that is, she considers that time perception is based on an abstract representation that is not tightly coupled with a particular sensory modality).

Pietrini et al. (2004) designed a study to test whether the information about faces and object categories that is represented in the ventral visual pathway by distinct patterns of neural activity is strictly visual or a more abstract, supramodal representation of object form. Using fMRI, the authors measured patterns of neural responses evoked during visual and tactile recognition of faces and manmade objects in sighted participants and during tactile recognition in a group of blind participants. Their results were taken to demonstrate that the representation of objects in the ventral visual pathway is not simply a representation of visual images, but, rather, is a representation of more abstract features of object form. Tamber-Rosenau et al. (2013) came to similar conclusions demonstraing that specific regions of the prefrontal cortex (i.e., dorsolateral prefrontal cortex and anterior insula) are insensitive to sensory modality or, at least, significantly less modality-sensitive than other brain regions. One can also think of the perception of a person’s identity or sex/gender as amodal (Awwad Shiekh Hasan et al., 2016; Kamachi et al., 2003; Smith et al., 2007).

There has also been interest from cognitive neuroscientists in trying to understand which, if any, brain regions represent amodal conceptual knowledge (e.g., Fairhall & Caramazza, 2013; Machery, 2016; Pobric et al., 2010; see also Van Doren et al., 2010). Though, as was noted in the *Introduction*, the debate between the modalists and the amodalists has reached something of a standstill. One potential solution from Michel (2021) builds on Patterson and Lambon Ralph’s (2016) hub-and-spoke hypothesis of semantic memory. In particular, Michel has argued that we should give up on a neat dichotomy between modal and amodal representations, and instead go for a graded solution, using the Predictive Processing framework model: “In this model, a concept is a distribued multi-level network of concept units. A specific tokening of a concept can include, context-dependently, nodes from all across the hierarchy, from peripheral sensorimotor areas to the highest cortical levels….In sum, in this view, there are no separate modal and amodal systems of representational structures in the brain; modality and amodality correspond to limiting cases of the (context-sensitive) processing depth in a distributed, hierarchical concept network.” (Michel, 2021, p. 673).

The aim amongst a number of cognitive neuroscientists would therefore appear to be try and localize the intervening central stages of information processing that map sensation to behaviour. They talk of amodal function, and amodality would appear to be functionally defined. Importantly, this use of the term ‘amodal’ stands apart from the way in which the term has been used by developmental psychologists (when talking about ‘amodal relations’). However, given that specific brain areas are never activated in isolation and that networks of activation involve the unisensory cortical areas, one might question whether there is ever such a purely amodal process, at least when referring to perceptual input. Michel’s (2021) context-dependent approach to treating amodal-modal as a continuum, rather than a strict dichotomy, may help our conceptualization here. Additionally, amodalism has been a recent dominant view in cognitive psychology, in connection with the surge of the computational view of the mind, and especially with Fodor’s (e.g., 1975) “Language of Thought” thesis. Recently, modalist positions have also resurged strongly (some call it “neo-empiricism”) in the context of the embodied cognition paradigm, which stresses that our conceptual apparatus is being shaped by the constraints of our body and sensory apparatus (e.g., Clark & Chalmers, 1998; Lakoff & Johnson, 1999).

While modal views characterize conceptual representations as states corresponding to ‘re-enactments’ or ‘simulations’ of sensory or motor states involving the sensorimotor areas of the brain, amodal representational systems (e.g., Fodor, 1975; Pylyshyn, 1984), in turn, are not associated with a specific modality. They are formal, language-like and ‘abstract’, and their symbols are processed syntactically, i.e., in virtue of some formal aspects (not meaning or content). Such representational systems work roughly like a formal calculus of symbols, much like a natural or formal language consisting of syntax/grammar and word forms (see Calzavarini, 2023, for a recent discussion of amodality in neurosemantics).

## Perception science: Amodal completion

First appearing in the 1950s (see Glynn, 1954; Kanizsa, 1954, 1970), ‘amodal completion’ is a phrase that is frequently used in the perception science literature when talking about perceptual completion (e.g., of occluded stimuli; e.g., Kanizsa & Gerbino, 1982; Kim et al., 2014; see Gerbino, 2020, for a recent review). Gerbino (2020, p. 12) defines amodal completion as “the amodal extrapolation/interpolation of proximal parts to generate a perceptual entity consistent with the object model evoked by them”. Nanay (2018, p. 1) proposed that amodal completion should be defined as “the representation of those parts of the perceived object that we get no sensory stimulation from”.^17^ In the context of its original formulation, amodal completion has often been defined in contrast to modal completion, with the former referring to the perceptual completion of occluded objects, and modal completion the perceptual completion taking place in the foreground (Gerbino, 2020).

The majority of the literature on amodal completion is found within vision science (e.g., Kanizsa, 1979; though see Gallace & Spence, 2011, for a review of the evidence supporting tactile/amodal completion). Famous examples of amodal completion in the visual modality, such as, for example, the Kanizsa triangle, are completed in the absence of any direct sensory input from the amodally completed regions. It can be argued, though, that the object that is completed is in a very real sense *visual* (i.e., it has nothing to do with smell, taste, sound, touch, etc).^18^ Relevant here, it is well documented that early cortical processing in the case of amodal completion and processing has even been detected in the primary visual cortex (see Bakin et al., 2000; Ban et al., 2013; Komatsu, 2006; Scherzer & Ekroll, 2015; Thielen et al., 2019).

Such a characterization of amodal completion can be traced back to related notions from early phenomenological authors, such as Husserl’s (Husserl, 1991, 2001) ‘anticipation’ of perception or ‘protention’ (Almäng, 2014). Conceiving of perception as necessarily incomplete and partial, Husserl claimed that it always admits degrees of fulfilment (Husserl, 2001, pp. 205-206 and p. 383). Husserl went on to suggest that such fulfilment depends on the perceptual contents that are anticipated by the subject through protentional mechanisms, i.e., the ability to extend perception beyond the actual contents via expectation. When we perceive a tree, the tree is not entirely given in any one perception. As we move closer to (or farther away from) the tree or walk around it, other aspects of the tree manifest themselves: the texture of its trunk, its overall shape, its height (Husserl, 2001, pp. 7-8). Husserl described the dynamics of expectation and protention in perception as follows: “No matter how empty and indeterminate this anticipatory continuity may be, it cannot be completely indeterminate; the style, so to speak, of ‘what is to come’ is prefigured through what has just past […] Indeed, it is a primordial law that every retentional course […] immediately and steadily motivates and thus generates intentions of expectancy [i.e. protentions] that are determined in the sense of a similarity of style” (Husserl, 2001, p. 323).

The philosopher of perception, Walter Hopp, had the following to say concerning Husserl’s view: “As I perceive the table from here, I can see some of its parts and sides, while others are hidden from view. I am conscious not just of the seen parts of the table, but of the unseen parts as well, but emptily and indeterminately” (Hopp, 2011, p. 55). According to Husserl, therefore, some parts of the object are hidden from view, but they are nevertheless part of the act of (visual) perception. Consequently, if this view is correct, there are non-sensory data attached to each act of sensory perception. These non-sensory data are interwoven with the sensory data in order to constitute the content of the act of perception. Discussing the perception of the back of opaque solid bodies (involving the colorless continuation of the front surface into the occluded space), Michotte and Burke (1951/1962) evoked the notion of completion in a Husserlian way: “Apparently, the ‘visible’ part is *completed* by the ‘amodal’ presence of the posterior part of bodies and such presence, as well as the properties that characterize it, are determined by the structure of the ‘modal’ datum and, in the last analysis, by the system of visual excitations” (Michotte & Burke, 1951/1962; translated in Gerbino, 2020; though see also Anstis, 2018; Scherzer & Faul, 2019).

In his reflections on the philosophy of (amodal) perception, Bence Nanay (2018) echoes Husserl when he states that amodal completion “is part of our ordinary perception. It happens very rarely in real-life situations that we can perceive an object without exercising amodal completion: In natural scenes, there is virtually always occlusion because objects tend not to be fully transparent. Every time we see an object occluded by another object (which means every time we see anything in real life, barring odd cases of fully translucent visual scenes or very simple visual displays), we use amodal completion of the occluded parts of perceived objects (Bakin et al., 2000). And the same goes for the backside of any solid object – sometimes referred to as self-occlusion. Again, we do not receive any sensory stimulation that would correspond to the backside of solid three-dimensional objects, but there is nonetheless perceptual processing of this missing information – in a way reminiscent of amodal completion” (Nanay, 2018, p. 4).^19^

Despite the multidisciplinary and longstanding interest in the concept of ‘amodal completion’, the notion, as pointed out by Gerbino (2020, p. 19), remains “popular but tricky”. Difficulties arise from the ambiguities of the terms that are used to define the concept. In fact, ‘amodal’ is sometimes used in a phenomenological sense, to mark the absence of a perceptual stimulus/input that is nevertheless perceived, while the term completion is occasionally conceived of as a process of addition, integration, or fulfilment. Moreover, amodal completion has often been explained using related concepts such as mental imagery, visualization, and non-perceptual beliefs (see, e.g., Nanay, 2007). As such, one might therefore legitimately want to ask what, exactly, is ‘amodal’ in the case of ‘amodal completion’. At first sight, amodal indicates that no specific sense modality is involved in amodal completion, but still we are dealing with perception (e.g., common sensibles). By contrast, as already evidenced, the same concept has also been interpreted as indicating that amodal completion lacks sensory information to support the perceptual act. However, if it is not the presence of sensory information, it is at least the absence of a(modal) sensory information that seemingly prompts amodal completion. In Kanisza figures, for instance, the spatial configuration of the elements makes certain *visual* features absent, but not auditory or tactile ones. Hence, amodal perception is not amodal in the sense that it simply lacks modality, as it necessarily occurs with respect to a specific channel of sensations. This is in line with Gibson’s remark that amodal completion is genuinely perceptual: “The perception of occlusion, it seems to me, entails the perception of something which is occluded” (Gibson, 1972, p. 229). If this is true, than amodal completion is seemingly more properly related to the phenomenon of perceptual filling-in, which we now briefly examine.

### Amodal completion and perceptual filling-in

Amodal completion has been considered as an occurrence of filling in perceptual completion phenomena (Pessoa et al., 1998; van Lier & Gerbino, 2015; see also Overgaard, 2022). In other words, filling-in is a perceptual phenomenon whereby certain perceptual (especially visual, such as colour) features are perceived, though these features are not directly present to perception (on visual filling-in, see, e.g., Anstis, 2010; Komatsu, 2006; Weil & Rees, 2011). Unfortunately, just as in the case of amodal completion, the term ‘filling-in’ has been used in different ways by different people. Sometimes the term is used to describe what the subject perceives; sometimes it is used to refer to the (neuro)psychological processes underlying perception. The term is also used to describe different sorts of perceptual completion. For example, although illusory contours and brightness perception probably involve different neural processes, the concept of filling-in is often used in association with both: a line segment is said to fill in between the inducers, and brightness is thought to fill in across regions (Pessoa et al., 1998).

A major conceptual issue here would appear to depend on clearly distinguishing between the *content* of a representation and the *vehicle* or *medium* of that representation, i.e., the cause that triggered that representation. Seemingly, amodal perception is used with respect to the content of perceptual knowledge, which might not merely reflect sensory information gathered through sensory channels, i.e., the external cause (see Sorensen, 1999, 2007).^20^ A potentially interesting line of research would be to investigate those perceptual situations in which the observers fail to accomplish amodal completion or filling-in processes. For example, much of the effect of surreal paintings is based on the perceptual impossibility of the depicted scene, with the observer struggling to try to make sense of incoherent percepts, which can be neither completed nor filled in (e.g., see Hamer’s (Hamer, 2023) perceptual analysis of René Magritte’s “Le Blanc-Seing”).

## Reconceptualization

Based on the literature that has been reviewed here, we propose to group most of the different occurrences of the term ‘amodal’– namely amodal relations, amodal mental categories, and amodal cognitive processes – into the following three categories: (1) Physical stimulus properties; (2) Sensory attributes that share the same phenomenal quality; and (3) Mental categories/representation (see Fig. 1). Physical properties of the stimulus are those features that characterize the perceptual quality of the stimulus. While some of these are essentially modality-dependent, such as colour, others might be modality-independent or, at least, can be picked up by different senses, such as size and shape. The latter can be considered as amodal physical properties (while acknowledging the fact that perfect redundancy is never achieved). Sensory attributes are those attributes that define the quality of sensory information. When these attributes share the same phenomenal quality while being referred to sensory information afferent to different modalities, such as, for example, intensity, they can be considered as amodal. Finally, amodal mental categories refer to those abstract concepts that can be applied to sensory objects or percepts across different modalities, such as numerosity and temporal order. In contrast to the physical properties and the sensory attributes, they can be conceived in purely formal or symbolic terms, i.e., lacking sensory contents. By contrast, conceiving of pitch, size, or intensity in purely formal terms would have no meaning.

That said, amodal completion is the only phenomenon that would fall out of our reconceptualization. This is probably due to the fact that in amodal completion the term is used in an entirely different way compared to how it is used in the cognitive and developmental sciences; in the former, it is used to describe the way a cognitive/perceptual *process* is accomplished, in the latter it qualifies the nature of sensory/perceptual/mental *attributes* (see, e.g., Palmer et al., 1996; Scherzer & Faul, 2019).

Complicating matters somewhat, literature has provided evidence of amodal perception in non-human animals (see Ratcliffe et al., 2016, for a review). The empirical evidence demonstrates that a perceptual phenomenon that is similar to amodal completion in humans is present across several species, such as mice and birds (e.g., see Kanizsa et al., 1993; Tvardíková & Fuchs, 2010). On the other hand, comparative studies suggest that mappings across the senses, especially between audition and vision, might represent a basic feature of the vertebrate sensory system (e.g., see Loconsole et al., 2021, 2022, on chicks; Ludwig et al., 2011, on chimpanzees; Korzeniowska et al., 2022, on dogs; and Loconsole et al., 2023, on turtles). Finally, research has convincingly shown that many of the mental categories that have been considered as amodal in humans are widespread in the animal kingdom as well (see Vallortigara et al., 2010, for a review), with studies potentially extending these capabilites to invertebrates (e.g., Rodríguez et al., 2015, on spiders). These studies suggest that, at least the studied abilities, cannot be explained exclusively in terms of sensory information/processing, rather calling for a broader evolutionary explanation.

One of the implications of the discussion of the literature that has been outlined here is that there is no convincing evidence for the existence of amodal sensory dimensions, no evidence for absolute cross-modal mappings (as suggested by Smith, 1987; see *Absolute versus relative cross-modal relations* section). At the same time, however, it may still be the case that there are different kinds of cross-modal correspondence, and those that have been posited by certain researchers as amodal, such as stimulus intensity, may nevertheless be less subject to context effects than other classes of cross-modal correspondence that have been documented to date (Krantz, 1972). It is interesting to consider here how cross-modal correspondences involving stimulus intensity have been given a structural, or physiological, explanation (Spence, 2011; Spence & Di Stefano, 2023; Stevens, 1957; see also Nehrkorn et al., 2015) that might provide an alternative explanation for why intensity-based cross-modal correspondences behave differently from statistical or affective correspondences, say, in terms of their earlier appearance in human development (Lewkowicz & Turkewitz, 1980), or else in terms of their resistance to context effects (cf. Brunetti et al., 2018; Fields et al., 1984; Guellaȉ et al., 2019; Stevens & Galanter, 1957; Walker et al., 2012; Woodward et al., 1976; though see Eisler, 1963; Henion, 1971, on some complications with the purely sensory assessment of olfactory intensity). That said, it remains an open research question to determine whether the cross-modal matching of stimulus intensity is any less relative than pitch-based cross-modal correspondences, for example (see Spence, 2019),

## Conclusions

In conclusion, the research that has been reviewed here clearly highlights the fact that the term ‘amodal’ has been taken up by several different groups of researchers over the years (see Table 3 for a complete list of relevant sources published since 1970). On the one hand, developmental researchers have been intrigued by the possibility that amodal relations might help to scaffold multisensory development (see Bremner et al., 2012). However, a closer look at the developmental literature soon reveals that there are at least three different definitions that variously refer to sensory and abstract properties of stimuli (see Table 1). The term has been used by developmental psychologists to refer to attributes such as, for example, the size and shape of an object, that are considered to be amodal because multiple senses (vision and touch) can potentially provide information relevant to the same physical stimulus/property (interestingly, however, only developmental researchers appear to describe this as such). Other sensory attributes such as, for example, stimulus intensity, have been considered by many researchers to be amodal because the same perceptual attribute is putatively picked up by multiple senses, even though this perceptual quality is associated with different modality-specific attributes (i.e., different kinds of physical/chemical stimulation).^21^ The third use of the term amodal by developmental researchers is in relation to those judgements that are not directly (or at least not necessarily) sensory, such as, for example, judgements of numerosity, rhythm, synchrony, rate, etc.

**Table 3:** Summary table highlighting the increasing (and changing) usage of the term 'amodal' in academic titles published since 1970. (See Supplementary Table 1 for detailed listing and classification of individual studies on which these summary statistics are based.) The results were obtained using Scopus with the following parameters: Period: 1970-2023; Discplines: Psychology, Neuroscience; Keyword: 'Amodal' in title only. Number of sources associated with the different categorizations of amodal, namely Amodal relations, Amodal cognitive processes, and Amodal completion are highlighted. Notice both how the usage of the term amodal has increased over the decades, but also how the majority of the time when the term is used it is in the context of amodal completion

Decade	Categories of amodal			Unclassifiable or alternative usage
	Amodal completion	Amodal relations	Amodal cognitive processes	
1970s	1	-	-	-
1980s	4	-	-	-
1990s	23	2	5	-
2000s	37	6	11	-
2010s	33	4	20	1
2020s	7	1	4	2

The notion of amodal has been debated across cognitive science and psychology as well (see e.g., Chow et al., 2023; Nanay, 2018; Wajnerman Paz, 2019) and, in some cases, criticized, with some suggesting that the dichotomy between amodal/modal is inadequate and therefore has to be ‘overcome’ (Michel, 2021; Scherzer & Faul, 2019). Cognitive neuroscientists sometimes use the term amodal when referring to central cognitive processes, neural regions or neural representations with (apparently) no preferential sensory component, while perception researchers often discuss ‘amodal completion’, though, as we have seen, this usage is not entirely unproblematic. Ultimately, though, we argue that there is, as yet, no convincing empirical evidence to support the claim that amodal sensory qualities exist (see Cohen, 1934, and Mellers & Birnbaum, 1982, for specific versions of this claim that have appeared previously in the literature). At the same time, the review also suggests that the use of the term amodal would be more meaningful with respect to abstract cognition (i.e., amodal mental categories) rather than sensory perception (i.e., physical stimulus/property or perceptual attribute), the latter being more adequately explained/understood in terms of cross-modal correspondences. The implication behind the use of the terms amodal and cross-modal would appear very different. By arguing that what have been termed amodal sensory dimensions might be better conceptualized as highly-redundant cross-modal correspondences, our aim is to challenge the very possibility of picking-up perfectly redundant sensory information across two or more senses. As the same time, the authors accept the possibility that non-sensory dimensions (e.g., rhythm, timing, duration…) might fruitfully be referred to as amodal. However, if this is the case, one would be eventually pulled back to the key question, which goes beyond the scope of this review, of how and why such amodal, non-sensory, mental categories are related to the sensory information they help to organize.

Finally, the term ‘amodal’ has been used in the perception science field to define a broad range of perceptual phenomena in which observers complete proximal parts of perceived objects that they get no direct sensory stimulation from. However, in the literature on perceptual completion, the term ‘amodal’ is seemingly used to describe the way in which a cognitive/perceptual *process* is accomplished, rather than, as it happens in developmental science and cognitive psychology, to qualify the nature of sensory/perceptual/mental *attributes*.

## Supplementary Information

Below is the link to the electronic supplementary material.

Supplementary Table 1 (Xlsx 34.1 KB)

## Footnotes

1 While amodal literally means ‘without modality’, the term cross-modal is typically used by scientists to highlight the link between one sense and another, or the influence of one sense upon another (see Spence, 2011, 2012). Thinking of the terms ‘amodal correspondence’ and ‘cross-modal correspondence’, the latter term would appear to imply that related, but different, stimuli, attributes, or dimensions are linked between the senses, whereas the former term carries with it the implication that it is the same fundamental stuff (or quality) that is being picked up by the different senses.

2 Though, intriguingly, Fowler’s (2004) chapter is the only one out of 54 chapters in the multisensory volume in which it appears to include the term ‘amodal’ in its title.

3 We return to this idea when we discuss the developmental psychologists’ reference to ‘amodal relations’.

4 These dimensions are also referred to as ‘supramodal’, ‘supermodal’ and ‘intermodal’ (e.g., see Calzavarini, 2023).

5 In particular, at one point Von Hornbostel (1927/1950, p. 214) writes that: “What is essential in the sensuous-perceptible is not that which separates the senses from one another, but that which unites them; unites them among themselves; unites them with the entire (even with the non-sensuous) experience in ourselves; and with all the external world that there is to be experienced.”

6 Eleanor Gibson (1983) argued for the same position in the context of phylogeny, namely, in the development of the individual. Referring to human development, she argues that: “The perceptual systems work together from the start” (p. 25) and that “modal properties, like color, are … differentiated or dissociated from the whole, which is more primitive” (p. 38).

7 The argument here is based on the idea that information across the senses is integrated. This suggestion presupposes that information is initially separate and is then subsequently integrated at some point during perception. However, it should be noted that such a perspective is not held by all. For example, the Gibsons, as well as those developmental researchers who have been inspired by them (e.g., Bahrick, Walker-Andrews), have instead argued that amodal properties are perceived directly instead of being integrated across the senses. That is, Bahrick and Lickliter do not consider that the perception of amodal properties involves multisensory integration. In contrast, the IRH is consistent with a Gibsonian approach to perceptual development, according to which, learning occurs through the differentiation of information, that observers detect invariants in their environment, and that information is already unified.

8 Elsewhere, Marks et al. (1987, p. 5) talk of the “perceptual, cross-modal equivalence with respect to intensity”. Here, there is an intriguing contrast with the pick up of frequency or vibration, where for a certain range of frequencies, it is possible to both hear and feel the same physical stimulus. However, even though the same physical properties of the stimulus are being picked up, their phenomenal quality is very different, giving rise to the phenomenal quality of pitch only in the auditory case. What is more, cross-modal matching studies reveal that the same physical frequency of vibration is typically not matched by participants (Björkman, 1967), arguing against Smith’s (1987) notion of an absolute correspondence (see also Marks, 1995; Pepermans & Corlett, 1983).

9 It is important to note the debate here is much more important than merely terminological (cf. Stein et al., 2009).

10 This is partially in line with Bahrick’s (Bahrick, 2010, p. 44) claim that: “Amodal information includes changes along three basic parameters of stimulation – time, space, and intensity.” However, one important difference is that despite time and space having no sensory marker, intensity has a sensory signature.

11 She writes that “Information for [amodal features] may be extracted from more than one kind of sensory experience. Perhaps all cross-modal similarity is amodal in that sense. We need, however, to remind ourselves that these similarities do not necessarily require an explanation based on association” (Gibson, 1969, p. 219). See also Di Stefano and Spence (2023) for a theoretical discussion of perceptual similarity across the senses.

12 According to such a definition of amodal, one might also perhaps want to consider cross-media artistic styles as being amodal mental categories that help to organize, or think about, perceptual matter (e.g., Arnheim, 1976, 1986; Dailey et al., 1997; Duthie & Duthie, 2015).

13 Roy Sorensen goes even further, saying that sensory stimulation is, at least in some cases, not even necessary for perception (Sorensen, 1999, 2007).

14 It is also worth noting here that these considerations can be interestingly related to the debate on abstract concepts, in the sense that the cross-species existence of amodal categories such as numerosity might challenge embodied theories of abstract concepts, based on language (i.e., metaphors), emotion, or sensorimotor information (see Borghi et al., 2017, for a review). In fact, it would seem difficult to affirm that there is continuity between invertebrate or rats and humans with respect to even one of the above-mentioned factors.

15 Interestingly, though, Ernst and Banks (2002) make no mention of amodal in their paper.

16 Though note that in the case of rate perception it is not possible to individuate the elements, bringing to mind Leibniz’s (Leibniz, 2015, pp. 23-27) famous distinction between clear and distinct ideas (numerosity, I discriminate 2 vs. 3 and can say why/how much) versus clear and confused ones (I discriminate faster vs. slower but cannot say exactly why/how much).

17 Note, however, the fact that by evoking the ‘umbrella’ concept of representation, Nanay’s (2018) definition would likely require additional clarification.

18 At the same time, however, it should be noted that tactile completion presumably also occurs whenever we feel an object between our fingers, for example (Gallace & Spence, 2014; O’Regan, 1992).

19 Elsewhere, one can find talk of ‘amodal wholeness’, defined as “the vivid percept of object unity and wholeness that occurs even though the observer does not actually see a contour in regions where completion of the whole object takes place at a level above the ‘happening’ ” (Pinna & Albertazzi, 2011, p. 335).

20 Given these issues, one might wonder whether amodal completion or perceptual filling-in occur, for instance, in the case of multisensory flavour perception (cf. Mausfeld, 2010). As Hartshorne (1934, p. 205) notes when considering the possibility of flavour Gestalts: “The problems of gustatory preference involve the same principles of harmony, contrast, and dissonance which explain beauty in the higher senses” (see also Spence, 2015; Spence & Di Stefano, 2022a).

21 We believe that those stimulus dimensions considered under these first two definitions may be better conceptualized as highly redundant cross-modal correspondences (see Parise & Spence, 2013) rather than necessarily as amodal sensory dimensions (Spence & Di Stefano, 2023).
